# A Multi-center Gadolinium-ethoxybenzyl-diethylenetriamine Pentaacetic Acid (Gd-EOB-DTPA) MRI Dataset with Expert Annotations and clinicopathological data

**DOI:** 10.1038/s41597-026-07483-x

**Published:** 2026-05-27

**Authors:** Tingting Xie, Dongping Ke, Demin Xu, Ziwei Liu, Zixuan Hua, Tianqi Zhu, Chunlan Huang, Xunqi Li, Ruibin Huang, Cheng Lu, Zhendong Luo, Zaiyi Liu

**Affiliations:** 1 Guangdong Cardiovascular Institute, Guangdong Provincial People’s Hospital, Guangdong Academy of Medical Sciences, Guangzhou, 510080 China; 2 Department of Radiology, Guangdong Provincial People’s Hospital, Guangdong Academy of Medical Sciences, Southern Medical University, Guangzhou, 510080 China; 3 Guangdong Provincial Key Laboratory of Artificial Intelligence in Medical Image Analysis and Application, Guangzhou, 510080 China; 4 Medical imaging center, Peking University Shenzhen Hospital, Shenzhen, 518036 China; 5 Department of Radiology, The First Affiliated Hospital of Shantou University Medical College, Shantou, 515041 China; 6 Department of Radiology, Shenzhen Shenshan People’s Hospital, Shenzhen, China; 7 Medical Research Institute, Guangdong Provincial People’s Hospital, Guangdong Academy of Medical Sciences, Southern Medical University, Guangzhou, 510080 China; 8 Department of Radiology, The University of Hong Kong - Shenzhen Hospital, Shenzhen, China

## Abstract

Liver resection is a cornerstone treatment for liver tumors, yet post-hepatectomy liver failure (PHLF) remains a severe and life-threatening complication with no effective treatment. Recent advances in artificial intelligence (AI) have shown promise in addressing this challenge; however, progress has been hindered by the limited availability of high-quality, expert-annotated, multi-center datasets. To bridge this critical gap, we present a multi-institutional dataset of preoperative Gd-EOB-DTPA-enhanced MRI scans, comprising 14,895 images from 220 patients across three academic medical centers. This comprehensive dataset includes 22,342 expert annotations of key anatomical structures (i.e., liver, Couinaud segments, liver tumors, spleen, and psoas muscle), detailed clinicopathological variables, and rigorously adjudicated PHLF outcomes. We also release U-Net-based automated segmentation tools to support reproducible region-of-interest delineation. Furthermore, we provide an illustrative (non-validated) pipeline demonstrating how FLR volumetry, MRI-derived functional parameters, and clinical risk factors can be integrated to support downstream analysis and methodological development. The described pipeline is illustrative and does not represent a validated predictive model. This dataset aims to facilitate research in FLR assessment and PHLF prediction, providing a resource for research on preoperative assessment.

## Background & Summary

Liver resection remains a cornerstone in the management of patients with benign and malignant liver tumors^1–3^. Despite advancements in surgical practice, post-hepatectomy liver failure (PHLF) continues to be a major cause of morbidity and mortality following liver resection^4,5^. Accurate preoperative prediction of PHLF is therefore critically important for candidate selection and surgical planning.

Current clinical practice typically relies on CT-based multiplanar and 3D reconstructions to assess future liver remnant (FLR) volume prior to hepatectomy, owing to their reliability and ease of implementation^4,6,7^; however, FLR volumetric assessment alone is often insufficient for predicting functional hepatic reserve, particularly in patients with underlying liver disease or those who have received preoperative chemotherapy. Both the volume and functional capacity of the FLR are critical determinants in preoperative assessment^8,9^. Gadolinium-ethoxybenzyl-diethylenetriamine pentaacetic acid-enhanced magnetic resonance imaging (Gd-EOB-DTPA-enhanced MRI), a modality utilizing a hepatobiliary contrast agent selectively taken up by hepatocytes, has emerged as a valuable tool in the management of liver diseases^10–12^. In preoperative planning, Gd-EOB-DTPA-enhanced MRI plays a dual role by providing high-resolution anatomical imaging for accurate FLR volumetry while simultaneously enabling functional assessment through hepatocyte-specific contrast uptake during the hepatobiliary phase (HBP)^4^. This combined volumetric-functional capability makes it an ideal modality for comprehensive preoperative one-stop FLR assessment and prediction of PHLF. Consequently, AI models trained on Gd-EOB-DTPA-enhanced MRI data may offer advantages over those based on CT or conventional MRI because they can integrate both anatomical and functional imaging features. Such integration provides a more physiologically grounded and potentially more accurate foundation for PHLF prediction.

However, the development of automated, quantitative methods for FLR assessment and PHLF prediction has been hampered by the lack of high-quality datasets. Most currently available public datasets rely on CT imaging and clinical information^13,14^, which limits the ability to achieve concurrent quantification of both FLR volume and function prior to liver resection. To date, there remains no publicly accessible dataset based on Gd-EOB-DTPA-enhanced MRI that enables simultaneous evaluation of FLR volume and functional capacity. Most publicly available imaging datasets have primarily focused on CT-based volumetry or tumor detection, with limited integration of functional imaging and PHLF-specific outcomes. For instance, collections in The Cancer Imaging Archive (TCIA) and similar repositories offer valuable CT-based FLR or tumor segmentation data, but they generally lack 1) hepatobiliary-phase MRI for functional liver assessment; 2) expert annotations of key anatomical structures necessary for automated FLR volumetry and functional quantification (e.g., Couinaud segments, spleen, and psoas muscle); and 3) systematically curated clinicopathological variables paired with rigorously adjudicated PHLF outcomes. The absence of such integrated, multi-modal, and expert-annotated datasets has limited the development of robust, AI-driven tools for PHLF risk stratification.

**Fig. 1:**
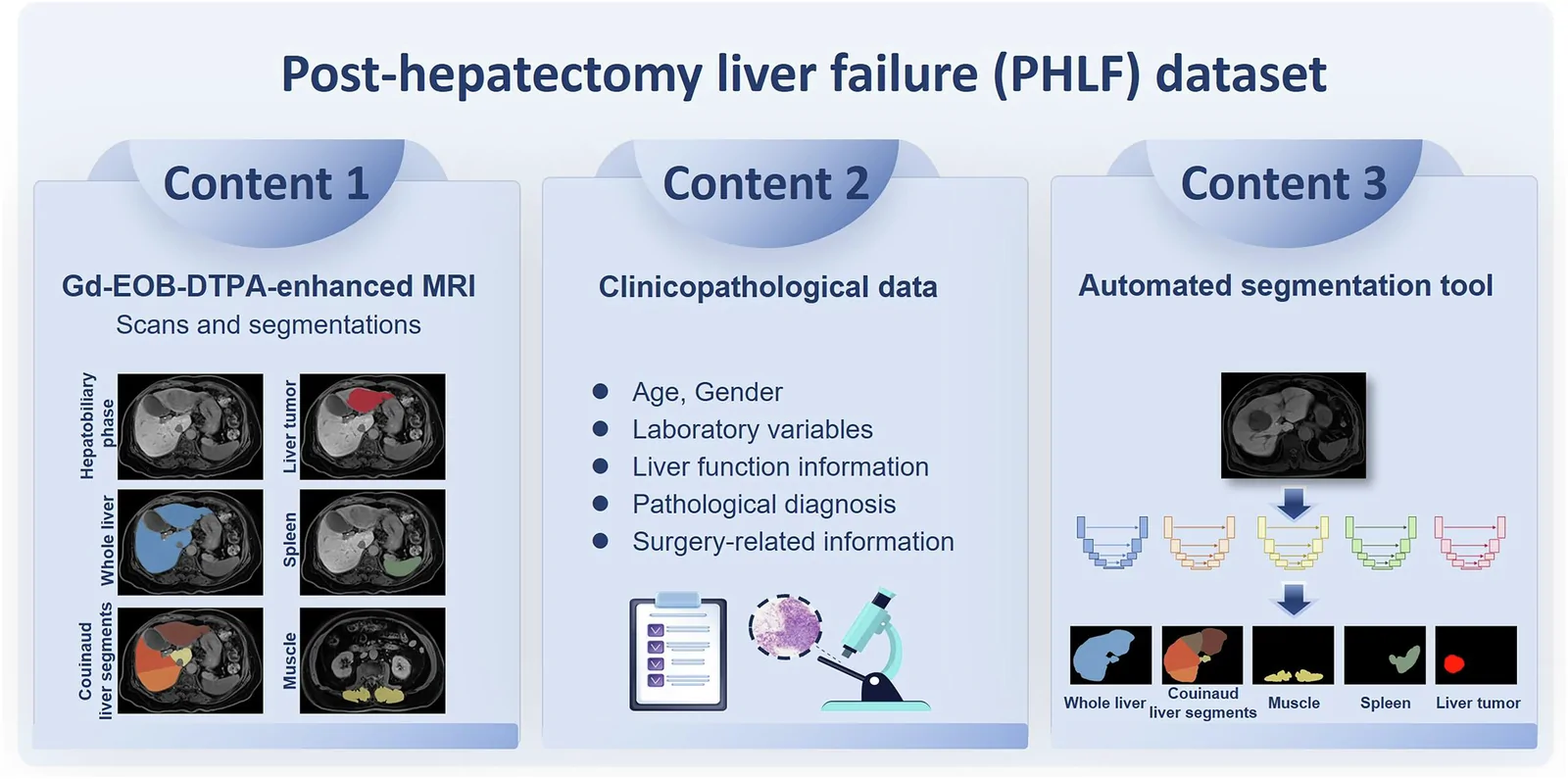
Overview of the PHLF dataset.

To address this critical gap, we present a multi-institutional dataset of preoperative Gd-EOB-DTPA-enhanced MRI scans collected from three academic medical centers. The dataset comprises: 1) comprehensive expert annotations of key anatomical structures—including the whole liver, Couinaud liver segments, liver tumors, spleen, and psoas muscle—enabling volumetric and functional quantification of the FLR; 2) detailed clinicopathological data supporting the evaluation of patient-related and surgery-related risk factors; and 3) a suite of U-Net-based automated segmentation tools, with each model specifically fine-tuned for distinct anatomical structures (e.g., liver, tumors, spleen), to facilitate efficient region-of-interest delineation and large-scale data analysis (Fig. 1).

This dataset represents the first multi-center resource of Gd-EOB-DTPA-enhanced MRI scans featuring comprehensive annotations specifically designed for the simultaneous assessment of FLR volume and function. It provides a unique foundation for quantitatively evaluating FLR-related risk factors, while the accompanying clinicopathological data enable assessment of patient-specific and surgery-related risks. By integrating these three critical risk dimensions, this dataset establishes a comprehensive platform for developing multimodal machine learning algorithms and imaging biomarkers aimed at improving preoperative risk stratification and clinical decision-making for candidates prior to liver resection.

## Methods

### Ethical statement

This retrospective study was approved by the Ethics Committee of Peking University Shenzhen Hospital [IRB numbers: 2025 (148)], the Ethics Committee of First Affiliated Hospital of Shantou University Medical College [IRB numbers: B-2025-253], and the Ethics Committee of the University of Hong Kong - Shenzhen Hospital [IRB numbers: 2025 (266)]. The approved research protocol explicitly stated that all patient data would be strictly anonymized and that the anonymized data may be released publicly. This protocol was reviewed and approved by all three IRBs. Given the use of anonymized patient data throughout the study and the retrospective nature, the requirement for informed consent was waived by the ethical standards outlined in the Declaration of Helsinki and its later amendments.

### Patient cohorts

Patients who accepted hepatic resections from the Peking University Shenzhen Hospital (center 1), the First Affiliated Hospital of Shantou University Medical College (center 2), and the University of Hong Kong - Shenzhen Hospital (center 3) between September 2016 and July 2025 were retrospectively collected consecutively.

**Fig. 2:**
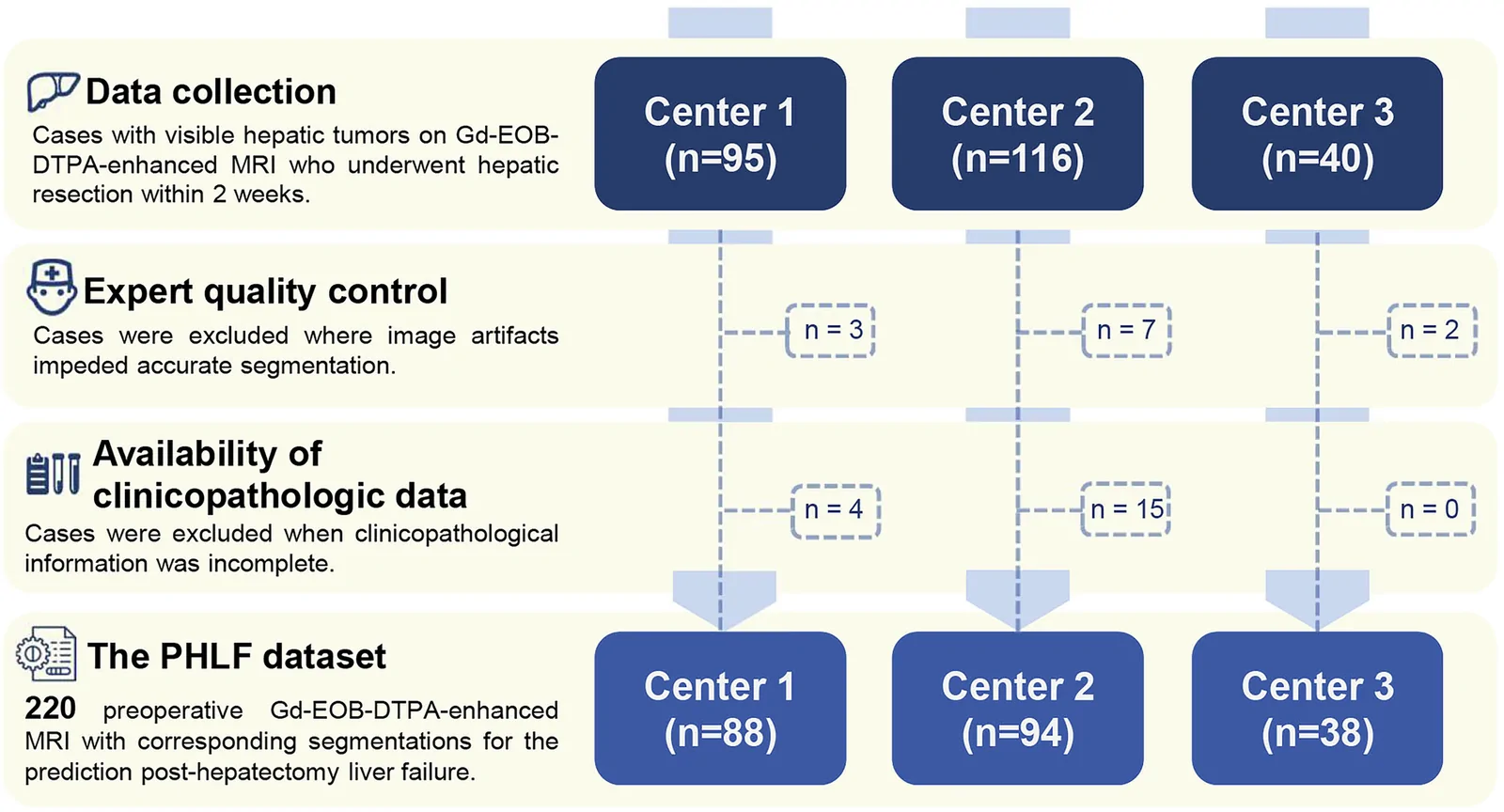
Flowchart of building the PHLF dataset.

The inclusion criteria are (a) patients with visible hepatic tumors on preoperative Gd-EOB-DTPA-enhanced MRI who undergo hepatic resection within 2 weeks. The following patients were excluded: (a) those with scans lacking complete liver coverage, unclear delineation of hepatic/portal vein branches, or substantial artifacts; (b) those with a history of hepatic resection, portal vein embolization (PVE), or associating liver partition and portal vein ligation for staged hepatectomy (ALPPS); (c) those with biliary obstruction and lack of hepatobiliary phase liver imaging; and (d) those with incomplete clinicopathological information. A detailed flowchart was demonstrated in Fig. 2. A total of 220 patients were included. All included MRI scans were restricted to the HBP; therefore, no adjustment for temporal shifts in imaging protocols was applied. Surgical practice was uniformly defined as hepatic resection, and the outcome definition (ISGLS grade B/C PHLF) remained consistent throughout the study period.

### Clinicopathological characteristics

Demographic, clinical, pathological, and laboratory variables were collected from electronic medical records in each center. It should be noted that the distribution of tumor pathology varies across centers, with Center 3 comprising exclusively hepatocellular carcinoma (HCC) cases—a pattern that may introduce center-specific bias and should be considered in subsequent analyses. Liver function-related variables, including the indocyanine green retention test at 15 min (ICG-R15)^15^, the model for end-stage liver disease (MELD) score^16^, the albumin-bilirubin (ALBI) score^17^, and pathology of nontumoral liver, and surgery-related variables, including resected tumor number, maximal tumor size, operating time, and blood transfusion in surgery, were summarized in Table 1.

**Table 1:** Clinicopathological characteristics of patient data.

Characteristics	Center 1 (n = 88)	Center 2 (n = 94)	Center 3 (n = 38)
Age (years) (median [IQR])	53 (42, 61)	61(54, 68)	59(48, 65)
Gender (%)			
Male	63(71.59)	67(71.28)	33(86.84)
Female	25(28.41)	27(28.72)	5(13.16)
ALT (U/L) (median [IQR])	27.00(19.00, 34.75)	28.08(19.76, 43.68)	31.90(24.60, 43.00)
AST (U/L) (median [IQR])	NA	31.21(23.45, 43.08)	31.70(25.90, 41.00)
Prothrombin time (sec)	13.35(12.90, 13.80)	11.00(10.40, 11.53)	13.55(12.98, 14.33)
INR (median [IQR])	1.01(0.97, 1.07)	0.96(0.91, 1.01)	1.07(0.99, 1.14)
ICG-R15 (median [IQR])	7.40(5.50, 11.28)	7.50(6.50, 8.90)	NA
MELD-Score (median [IQR])	7.00(6.00, 7.00)	7.00(6.00, 8.00)	8.00(7.00, 9.00)
ALBI-Score (median [IQR])	−2.65(−2.81, −2.48)	−2.71(−2.92, −2.39)	−2.81(−3.08, −2.50)
Pathological result (%)			
HCC	55(62.50)	71(75.53)	38(100)
ICC	4(4.55)	3(3.19)	0(0)
Hepatic metastasis	7(7.95)	8(8.51)	0(0)
Hepatic hemangioma	2(2.27)	1(1.06)	0(0)
Others	20(22.73)	11(11.70)	0(0)
Resected tumor number (%)			
Single	74(84.09)	82(87.23)	30(78.95)
Multiple	14(15.91)	12(12.77)	8(21.05)
Maximal tumor size[cm (mean ± SD)]	3.53 ± 3.09	4.88 ± 3.42	3.35 ± 2.88
Pathology of nontumoral liver (%)			
Hepatitis	16(18.18%)	1(1.06%)	0(0)
Fibrosis/Cirrhosis	39(44.32%)	40(42.55%)	35(92.11%)
Others	33(37.50%)	53(56.38%)	3(7.89%)
Surgery-dependent factors			
Blood transfusion in surgery [mL, median (IQR)]	0(0, 0)	0(0, 0)	0(0, 0)
Operating time [min, (mean ± SD)]	190.00(151.25, 255.00)	270.00(213.80, 328.50)	283.50(198.80, 387.30)
Major hepatectomy (%)	16(18.18%)	26(27.66%)	6(15.79%)
Minor hepatectomy (%)	72(81.82%)	68(72.34%)	32(84.21%)
Open hepatectomy (%)	31(35.23%)	31(32.98%)	34(89.47%)
Laparoscopic hepatectomy (%)	57(64.77%)	63(67.02%)	4(10.53%)
Kawaguchi classification Grade III	38(66.67%)	56(88.89%)	1(25.00%)
Post-hepatectomy liver failure grade B/C	5(5.68%)	5(5.26%)	6(15.79%)

Note. ALT, alanine transaminase; AST, aspartate transaminase; INR, International normalized ratio; ICG-R15, indocyanine green 15-min retention; MELD, model for end-stage liver disease; ALBI, albumin-bilirubin; HCC: hepatocellular carcinoma; ICC: intrahepatic cholangiocarcinoma; SD, standard deviation; IQR, inter-quartile range. Data are presented as n(%), median(IQR). NA indicates that the variable was not routinely recorded or available in the corresponding center’s clinical records. Major hepatectomy is defined as resection involving more than three Couinaud’s segments. “Others” under Pathological result (%) in Centers 1 and 2 includes benign or rare lesions difficult to distinguish from HCC/ICC on imaging: e.g., focal nodular hyperplasia, solitary necrotic nodule, perivascular epithelioid cell tumor, high-grade dysplastic nodule, hepatic angiomyolipoma, lymphoma, and teratoma. The Kawaguchi classification is a standardized grading system for assessing the technical difficulty of laparoscopic liver surgery, with Grade III denoting high-complexity procedures. Variations in data availability and distributions across centers reflect differences in clinical protocols and patient populations.

### MRI Acquisition

All Gd-EOB-DTPA-enhanced MRI were conducted using eight MRI scanners across three hospitals, performed at either 3 T (91.93%) or 1.5 T (8.07%). The dataset reflects real-world variability in scanner models and acquisition protocols across centers (detailed in Table 2). While this heterogeneity may enhance the generalizability of models trained on these data, users conducting intensity-sensitive analyses—such as radiomic feature extraction or signal-based functional assessment—are encouraged to apply appropriate harmonization methods (e.g., z-score normalization per scanner or histogram matching). Images are released without preprocessing to support flexible analytical approaches.

**Table 2:** Scanning parameters in all centers.

Category		Center 1(n = 88)	Center 2(n = 94)	Center 3(n = 38)
MR scanner	SIEMENS	55(62.50%)	94(100%)	38(100%)
	Philips	33(37.50%)	0(0)	0(0)
Sequence		hepatobiliary phase	hepatobiliary phase	hepatobiliary phase
Total number of MR slices (n)		6111	6156	2628
TR (ms)	4	55(62.50%)	94(100%)	14(36.84%)
	5	25(28.41%)	0(0)	24(63.16%)
	6	5(5.68%)	0(0)	0(0)
	others	3(3.41%)	0(0)	0(0)
TE (ms)	0	30(34.10%)	0(0)	14(36.84%)
	1	46(52.27%)	0(0)	24(63.16%)
	2	12(13.64%)	94(100%)	0(0)
	others	0(0)	0(0)	0(0)
Flip angle(degree)	9	17(19.32%)	41(43.62%)	13(34.21%)
	10	14(15.91%)	0(0)	2(5.26)
	20	11(12.50%)	0(0)	0(0)
	others	46(52.27%)	53(56.38%)	23(60.53%)
Slice thickness (mm)	3	56(63.64%)	93(98.94%)	23(60.53%)
	4	19(21.59%)	1(1.06%)	0(0)
	5	12(13.64%)	0(0)	0(0)
	others	1(1.14%)	0(0)	15(39.47%)
Slice gap (mm)	1.5	2(2.27%)	NA	NA
	2	19(21.59%)		
	6.5	12(13.64%)		
	others	55(62.50%)		
Number of excitations	1	88(100%)	94(100%)	38(100%)

### Expert segmentations

**Fig. 3:**
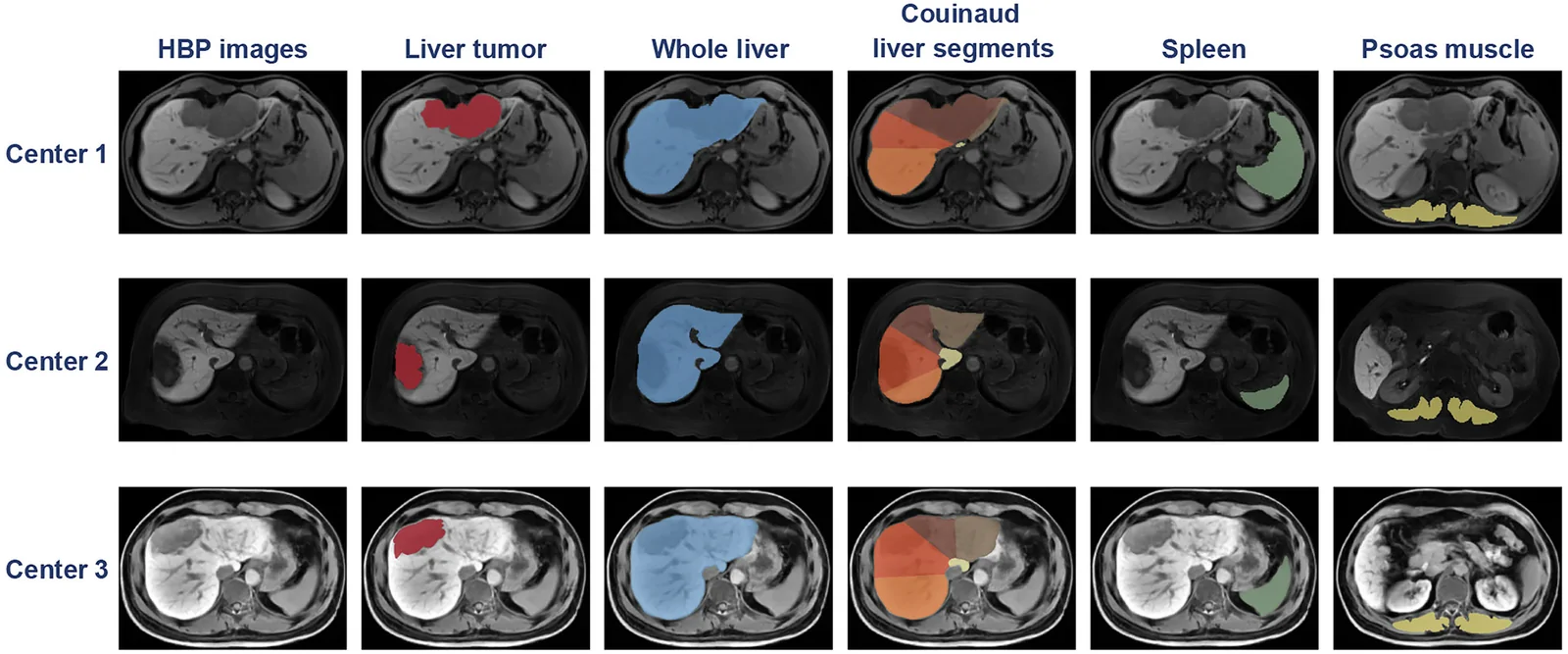
Example of hepatobiliary phase (HBP) Gd-EOB-DTPA-enhanced MR images and corresponding annotations. The first column shows HBP images acquired from the three centers. The second to the sixth columns illustrate the annotations of the liver tumor, whole liver, Couinaud liver segments, spleen, and psoas muscle, respectively.

The whole liver, liver tumors, Couinaud liver segments, spleen, and psoas muscle were delineated on the HBP Gd-EOB-DTPA-enhanced MR images. All annotations were made slice by slice on the 2D axial images by a radiologist with 5 years of experience (Dr. D.K) and subsequently reviewed, adjusted, and finalized by a senior abdominal radiologist with 15 years of experience (Dr. Z.L). To ensure inter-center consistency, the same two radiologists followed a standardized annotation protocol uniformly applied across all centers, which specified precise anatomical boundaries and consistent operational rules (e.g., handling of accessory spleens, exclusion of the main portal vein in liver annotation). Examples of HBP Gd-EOB-DTPA-enhanced MRI and annotations are demonstrated in Fig. 3.

All resected liver tumors are confirmed by pathology. In all cases, liver tumor annotations were provided exclusively for the resected liver tumors, irrespective of the presence of single or multiple tumors in the patient’s liver. Annotations of the whole liver, eight Couinaud liver segments, spleen, and muscle were provided for every case. The annotations were processed with ITK-SNAP version 3.8.0.

For whole liver annotation, the liver tumor, inferior vena cava, and main trunk of the portal vein were excluded, whereas vessels enclosed within the hepatic parenchyma were included. For spleen annotation, accessory spleens were included. For the psoas muscle, annotations were restricted to the region from the L3 vertebral body to the level immediately inferior to L3 within the scan range, as this proportion demonstrates a relatively larger cross-sectional area and is more appropriate for use as an internal reference standard. For Couinaud liver segment annotation, the two radiologists manually delineated segments using the Couinaud anatomical framework, which defines segments based on portal and hepatic vein branching patterns. On Gd-EOB-DTPA-enhanced MRI, these vascular landmarks are clearly visible on HBP images. Although segment boundaries are not directly visualized, radiologists reliably infer them using established anatomical reference points (e.g., gallbladder fossa, falciform ligament, main portal fissure). This methodology has been validated in previous studies^18–20^.

### Pipeline for automated volumetric and functional quantification of FLR prior to PHLF prediction

**Fig. 4:**
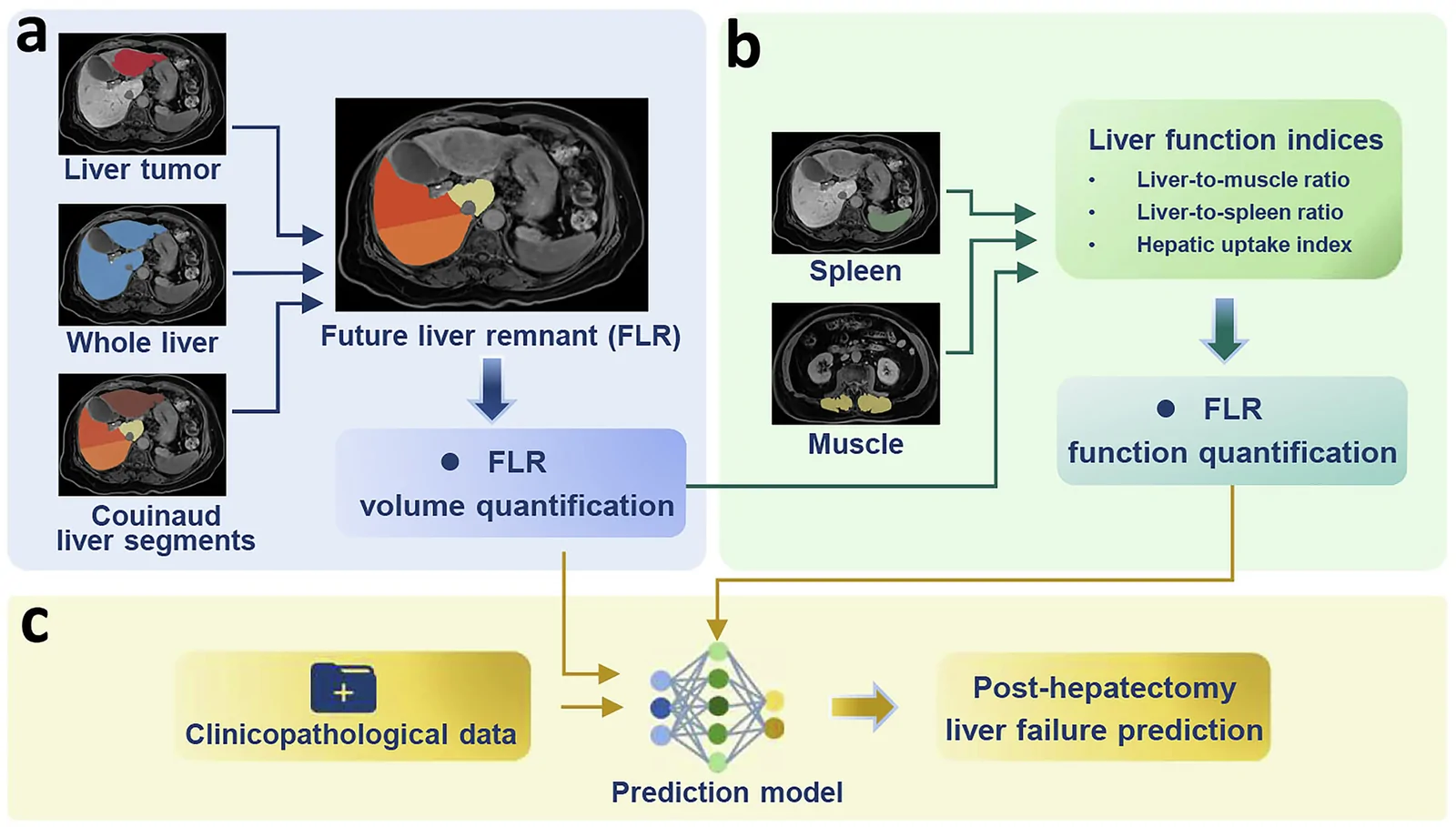
An example pipeline for automated future liver remnant (FLR) assessment and post-hepatectomy liver failure (PHLF) prediction. **(a)** Quantification of FLR volume based on annotations of the whole liver, Couinaud liver segments, and liver tumors, supporting automated segmentation of non-tumoral regions expected to remain after resection. **(b)** Quantification of FLR function through signal intensity–derived indices (e.g., liver-to-spleen and liver-to-muscle ratios) computed from delineated regions of the spleen, psoas muscle, and FLR. **(c)** Example of integrated risk stratification: a framework that combines volumetric, functional, and clinicopathological variables for multimodal PHLF risk evaluation.

This dataset enables a conceptual pipeline to demonstrate how FLR volume and function can be derived from the provided annotations and clinical variables. The described pipeline is illustrative and does not represent a validated predictive model (Fig. 4):

First, the FLR volume can be derived by measuring the volumes of Couinaud liver segments expected to remain after anatomical resection^5,21^. The provided annotations of the whole liver, liver tumors, and Couinaud liver segments enable volumetry of the FLR (detailed in Fig. 4a). The annotation of liver tumors provided critical information for tumor localization. When combined with annotations of the whole liver and Couinaud liver segments, this allows for automated detection, segmentation, and quantification of the FLR —specifically, nontumoral Couinaud liver segments, which are expected to remain.

Second, FLR function may be estimated using signal intensity-based liver function indices derived from Gd-EOB-DTPA-enhanced MR, such as the liver-to-muscle ratio, liver-to-spleen ratio, and hepatic uptake index, which are widely utilized^22–27^ (detailed calculation formulas are provided in Table 3). The annotated spleen and psoas muscle regions in the dataset support automated computation of these indices. When integrated with FLR volume, these metrics contribute to a comprehensive assessment of FLR function, representing a second critical dimension (i.e., FLR function) in PHLF risk evaluation (Fig. 4b).

**Table 3:** Summary of signal intensity-based methods for estimating liver function.

Indices	Formula
Liver-to-muscle ratio	SI_HBP_ of the liver/ SI_HBP_ of the muscle
Liver-to-spleen ratio	SI_HBP_ of the liver/ SI_HBP_ of the spleen
Hepatic uptake index	Liver volume × [(SI_HBP_ of the liver/SI_HBP_ of the spleen) - 1]

Note., SI, signal intensity; HBP: hepatobiliary phase.

Finally, by integrating clinicopathological data, FLR volume and liver function metrics, this multi-center dataset can serve as a foundation for developing integrated models that combine liver-associated factors (i.e., volumetric and functional parameters) with patient-specific and surgery-related risk factors, illustrating a potential approach to PHLF risk stratification (Fig. 4c).

### Outcome data

The outcome of interest was clinically significant PHLF, which was assessed for all 220 patients. The dataset includes a dedicated column flagging the occurrence of this outcome alongside other clinical and pathological variables. For this study, clinically significant PHLF was restricted to grades B and C as per the International Study Group of Liver Surgery (ISGLS) criteria^25^, thereby excluding grade A, which does not necessitate clinical intervention and carries a low mortality risk. The diagnosis of PHLF required (1) the concurrent presence, on or after the fifth postoperative day, of an elevated international normalized ratio (INR) > 1.2 and a total bilirubin level exceeding 22 μmol/L or the patient’s preoperative baseline; and (2) the condition must have warranted a clinical intervention, either non-invasive or invasive.

It should be noted that clinically significant PHLF (grade B/C) occurred in only 16 patients (7.27%) within this dataset. Given the relatively low number of positive PHLF events, this dataset is primarily intended as a resource for methodological development, model pretraining, and external validation, rather than as a standalone cohort for training high-capacity predictive models. Users are advised to consider this limitation and account for class imbalance when designing and interpreting model-based analyses. The higher incidence of PHLF at Center 3 (15.79%) may be attributable to a combination of more adverse patient baseline characteristics—namely, poorer hepatic functional reserve, 100% prevalence of HCC, and a higher proportion of multifocal tumors—and greater operative complexity, including longer surgery duration and a higher proportion of open hepatectomies^28^.

### Data De-identification

All personally identifiable information, as defined by Health Insurance Portability and Accountability Act (HIPAA) regulations, has been thoroughly removed from the medical images. The source Digital Imaging and Communications in Medicine (DICOM) files are processed into the Neuroimaging Informatics Technology Initiative (Nifti) format (with a.nii.gz extension) using the dcm2niix software tool (https://github.com/rordenlab/dcm2niix), which is configured to perform the anonymization during the conversion.

## Data Records

**Fig. 5:**
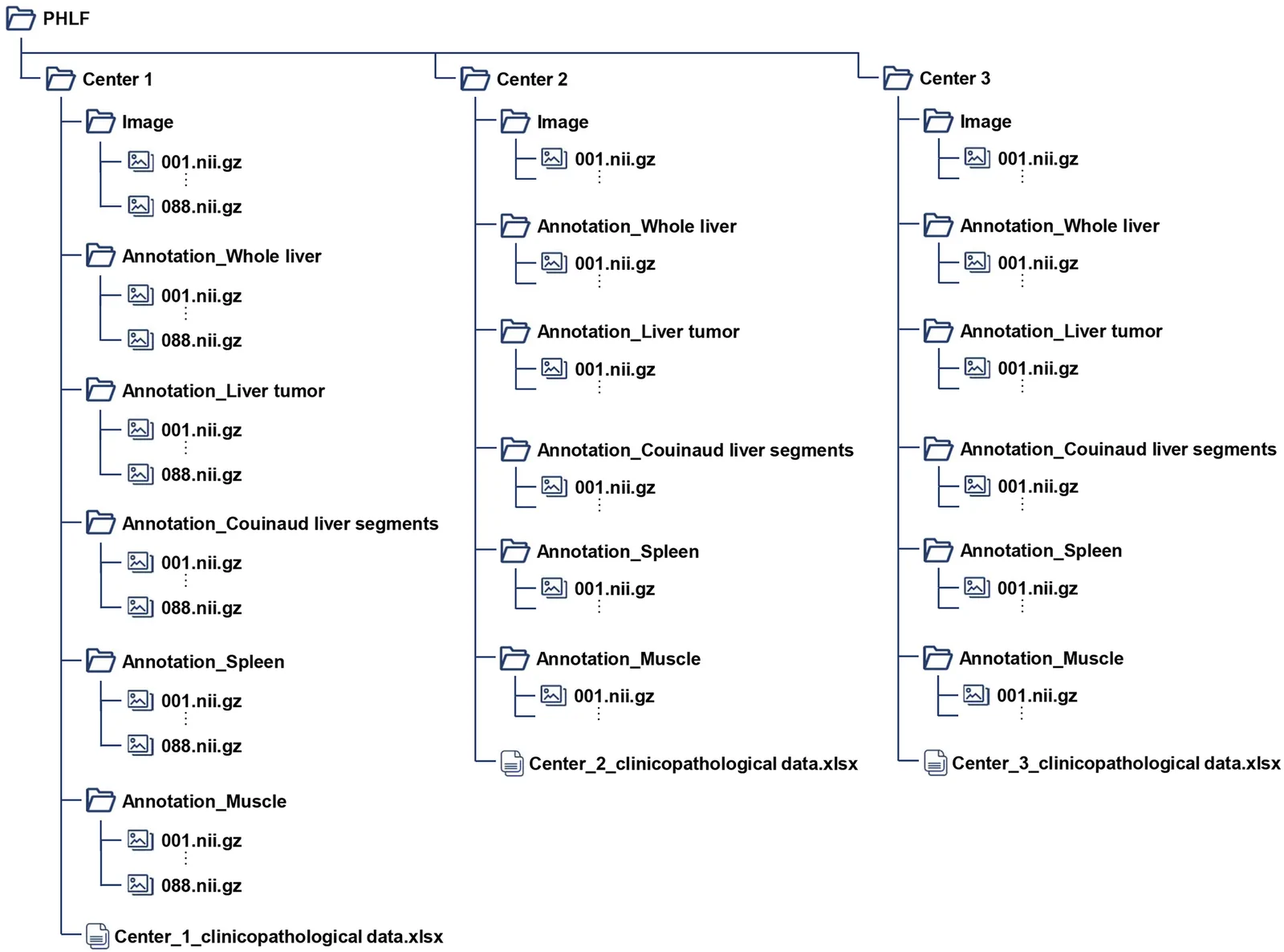
The PHLF dataset’s data structure, format, and nomenclature.

The dataset described in this work is publicly available on Zenodo^29^ (10.5281/zenodo.18622298). It includes the preoperative HBP Gd-EOB-DTPA-enhanced MRI and segmentation masks (whole liver, tumors, Couinaud liver segments, spleen, and psoas muscle) for all patients. Images and segmentations are stored in the Nifti format. Its structure (Fig. 5) is summarized as follows:

The root “PHLF” directory contains three subfolders, “Center 1,” “Center 2,” and “Center 3,” each housing the respective center’s HBP images.

Each center’s folder is systematically organized into:

1. An “Image” subfolder for the center’s HBP scans in Nifti format.
2. An “Annotation_Whole liver” subfolder for the corresponding whole liver segmentations.
3. An “Annotation_Liver tumor” subfolder for the corresponding liver tumor segmentations.
4. An “Annotation_Couinaud liver segments” subfolder for the corresponding annotations of Couinaud liver segments.
5. An “Annotation_Spleen” subfolder for the corresponding spleen segmentations.
6. An “Annotation_Muscle” subfolder for the corresponding psoas muscle segmentations.
7. An Excel file (Center_X_Clinicopathological data.xlsx) containing all clinicopathological data and outcomes, linkable to images via the “Patient-ID” variable.

The dataset deliberately provides images and segmentations without pre-computed radiomic features, allowing researchers the flexibility to apply their own extraction methodologies.

## Technical Validation

### Quality control for images

This dataset underwent rigorous quality assurance procedures at two distinct stages. First, subject eligibility was assessed based on predefined inclusion and exclusion criteria to ensure cohort homogeneity for subsequent analysis. Second, each MRI scan was individually reviewed to confirm the absence of significant artifacts and to verify adequate image clarity for accurate delineation of anatomical structures.

### Quality control for annotations

To ensure the quality of ground truth annotations, a rigorous, multi-step protocol was employed. Initial automated segmentations of the whole liver, liver tumors, Couinaud liver segments, spleen, and psoas muscle were generated using a U-Net-based model. To minimize potential bias from these initial AI outputs, the predicted masks were displayed with high transparency, guiding rather than dictating the subsequent manual refinement. These preliminary outputs then underwent a comprehensive two-tiered manual review process: first, a radiologist with 5 years of experience refined the AI-generated segmentations; subsequently, a senior abdominal radiologist with 15 years of expertise conducted a final quality check and performed additional refinements as required. The resulting consensus annotations, representing clinically adjudicated ground truth labels, are provided in this dataset.

### Inter-observer consistency assessment

To further validate the reliability of the manual annotations, inter-observer agreement was quantitatively evaluated between the two radiologists across all three participating centers. The Dice similarity coefficient (DSC) was computed for each annotated structure. The results, summarized in Table 4, demonstrate consistently high agreement, reflecting the robustness and reproducibility of the annotation protocol.

**Table 4:** Inter-observer agreement (Dice similarity coefficient, DSC) for anatomical structure annotations across three centers.

DSC	Liver	Liver segments	Liver tumors	Spleen	Psoas muscle
Center 1	0.93 ± 0.14	0.85 ± 0.14	0.92 ± 0.04	0.96 ± 0.01	0.89 ± 0.13
Center 2	0.90 ± 0.13	0.80 ± 0.13	0.94 ± 0.04	0.95 ± 0.01	0.93 ± 0.03
Center 3	0.97 ± 0.04	0.89 ± 0.08	0.92 ± 0.03	0.96 ± 0.02	0.93 ± 0.03

## Data Availability

The complete collection is accessible at Zenodo^29^ (10.5281/zenodo.18622298).

## Code availability

The automated segmentation tools can be accessed via https://github.com/Fangze-Guo/lxq_nnunet.git.
